# Baropodometric Assessment of the Podiatric Profile of Nursing Students in Clinical Settings: A Study Protocol

**DOI:** 10.3389/fpubh.2022.862048

**Published:** 2022-05-12

**Authors:** Rafael A. Bernardes, Sílvia Caldeira, Pedro Parreira, Liliana B. Sousa, Inês F. Almeida, Paulo Santos-Costa, Filipe Paiva-Santos, Arménio Guardado Cruz

**Affiliations:** ^1^The Health Sciences Research Unit: Nursing (UICISA: E), Nursing School of Coimbra (ESEnfC), Coimbra, Portugal; ^2^Centre for Interdisciplinary Research in Health, Institute of Health Sciences, Universidade Cattólica Portuguesa, Lisbon, Portugal

**Keywords:** foot health, ankle injuries, nurses, nursing, standing position, occupational health, baropodometric gait analysis

## Abstract

**Introduction:**

Nursing students are exposed to increased risks of developing foot and ankle disorders due to prolonged standing and walking positions during clinical settings. This can lead to high dropout rates from nursing degree, thus contributing to a future shortage in nursing professionals. This protocol aims to develop a study to understand the influence of prolonged standing and walking positions on nursing students' foot health, and specifically to study the relationship between the podiatric profile (regional force and pressure exerted on the foot) and related signs and symptoms.

**Methods and Analysis:**

A prospective observational cohort study will be conducted with 194 nursing students. Participants will be asked to walk through a baropodometric platform before and after a 5-month clinical training session. Assessment will focus on the change in podiatric profile, namely foot posture and foot function, at 5 months, and changes in foot health at 5 months. The study will start in January 2022 and it's expected to end by June 2022.

**Discussion:**

The study aims to perform an innovative assessment of nursing students' podiatric profile, which will allow for a comprehensive description of foot/ankle changes and their relationship with prolonged standing and walking contexts.

**Ethics and Dissemination:**

The study was approved by The Ethical Committee of the Health Sciences Research Unit: Nursing (UICISA: E), of the Nursing School of Coimbra (ESEnfC), with the approval code nr. P799_07_2021. The study was also recorded in ClinicalTrials.gov on the number NCT05197166. Findings will be used to publish articles in peer-review scientific journals and oral communications and posters at scientific meetings.

## Introduction

Nurses are one of the healthcare professionals most exposed to occupational health risks, mostly due to prolonged standing and walking contexts (1, 2). Evidence has suggested that prolonged standing is associated with the development of adverse health outcomes (3), particularly lower-limb disorders. One of the most common disorders concerns the ones in the foot/ankle region (4, 5).

Stolt et al. (6) state that prolonged standing is considered the second work-related factor hindering foot health, increasing the risk for the development of significant disorders, and affecting the quality of life. However, these disorders are a large and often unrecognized group of diseases (4, 6) having poor evidence based on the scarcity of related research. As so, the biomechanics of many foot disorders remain poorly understood (7), particularly the influence and relationship between biomechanical variables and external stressors, such as, prolonged standing or walking positions.

According to Bakker et al. (8), the increase in physical demands while in clinical settings is an important contributor to nursing students' late-dropout, thus contributing to the future professional shortage. Data from a recent prospective cohort study (9) shows that musculoskeletal complaints, including foot/ankle disorders, accounted for 39.9% of intention to leave nursing education and an actual dropout rate of 3.4% among nursing students. In fact, many authors state that the foot and ankle regions are the most commonly affected and reported locations for the development of disorders in nursing students (10–14).

In this sense, to effectively prevent future disorders, and for an adequate foot self-care promotion among nurses and nursing students, a suitable evaluation and description of the podiatric profile are necessary. Furthermore, a categorization of the most important foot/ankle variables that are affected by standing environments, as defined by Bernardes et al. (15), as potentially aggressive contexts in the nursing profession, often implying continuous static-bound positions (while standing) or continuous dynamic movements (with long built-up walking distances throughout the shift time), is deemed important for the development of preventive interventions.

An evaluation and assessment of foot and ankle dynamic variables, while in static or dynamic positions, can be performed by various means. One of the most reliable method to determine the podiatric profile and respective variables, namely plantar pressure, is baropodometry (16). It's usually used to analyze the pressure areas exerted by the body in both motion and static positions, providing a dynamic gait analysis, distribution of loads during walking, peak pressures, and contact time with the ground, and also detection of areas in risk on the foot. Many previous studies have used plantar pressure assessments to identify foot pathologies and risk factors (17), with important results, namely detecting altered regional loadings (18), significant differences in foot kinematics (19) and pronated foot function (20).

Therefore, this paper aims to describe a protocol for a prospective observational study on the influence of prolonged standing and walking positions on nursing students' foot health, and relationships between the podiatric profile (regional force and pressure exerted on the foot) and related signs and symptoms.

## Methods and Design

### Study Design and Setting

A prospective observational cohort study will be conducted between January 2022 and June 2022 in a Nursing public school in Portugal. Data collection will take place at the end of January and in the first days of February.

### Participants and Recruitment Process

The study will enroll nursing students from a Portuguese Nursing School, and which are exposed to standing environments during acute clinical settings (e.g., hospitals). The recruitment process will be developed according to the following inclusion criteria: (i) nursing students enrolled, at the moment of the study, in a Nursing degree; (ii) nursing students that, at the moment of the recruitment phase, are not enrolled in a clinical learning setting; and the following exclusion criteria: (i) diagnosed chronic systematic diseases (e.g., rheumatoid arthritis); (ii) diagnosed metabolic disorders; (iii) visible lower limb swelling; (iv) presence of contraindications for baropodometric-related measurements; (v) history of orthopedic neurological and/or musculoskeletal problems likely to affect gait; and (vi) students that, at the moment of the recruitment phase, are also committed to a professional working activity or involved in high competition sports, which might influence foot health (e.g., waitress, door-to-door delivery, among others).

Participants will first be addressed in a project presentation session, where informed consent will be provided for their analysis and signature. Subsequently, the days for data collection will be scheduled, taking into consideration the most convenient periods for students and the research team. In this sense, a convenience sample will be recruited, as students will voluntary show up to participate in the study. This sampling method was chosen, rather than randomization, due to time constraints regarding project schedule, and also the need to achieve a representative sample. As we are performing the study only in one Nursing School, due to researchers' availability and ethical approvals, randomization could include students that wouldn't be able or didn't want to be part of the research in progress. In this sense, the research team opted to include volunteers, also helping decrease potential loss to follow-up.

In the Nursing School involved in this study, clinical training has an average duration of 4 to 5 months for students in the third and fourth year of the Nursing degree. Thus, the study will depend on this timeframe for the respective follow-up period.

According to the defined criteria, all undergraduate nursing students at the third year will be recruited as participants. Each academic year, an average of 300 students are enrolled, and for a confidence interval of 95% and a margin of error of 5%, the sample size needed is 169 participants. Also, an a priori 0.05 significance level is defined and a response distribution of 50% is assumed. To prevent potential losses, we've added an additional 15% of the total sample size, thus aiming to recruit 194 students.

All data collection will take place inside the Nursing School, in specifically chosen places, previously tested for the study, and will be performed by two researchers, which will be different from those responsible to analyze data.

### Exposures, Outcomes and Confounders

The exposure under study consists of a particular environment that poses particular biomechanical risks to the foot/ankle region. According to Bernardes et al. (15), standing environments include those of prolonged standing and prolonged walking, which can be defined as spending at least 5% of occupational time standing or walking (21).

The first primary outcome of interest is related to the change in podiatric profile at 5 months and will be evaluated at month 0 and month 5, being an objective and quantitative outcome. In this case, specific foot-related variables while walking will be recorded, illustrating the actual behavior of the foot during activity. The assessment of the podiatric profile follows the important premise that the medial longitudinal arch is one of the most important and highly variable structural characteristic of the human foot (22).

The relevant variables that describe this outcome are summarized in Table 1.

**Table 1:** Specific podiatric profile variables.

**Category**	**Variables**	**Definition**
**Kinematic variables**	Forefoot width/spreading	Distance, in millimeters, between two straight lines perpendicular to the Chopart joint and tangential to most medial and most lateral points of the heads of first and fifth metatarsals, respectively.
	Foot angle at contact (sagittal plane)	Dorsiflexion angle at contact, in relation to the sagittal plane.
	Foot eversion	Or pronation, is an angular movement where the foot moves away from the medial plane.
	Foot adduction	Is the angular movement were the foot moves toward the medial plane.
	Foot external rotation	Rotation of the joint away from the midline, measured as an angle.
	Ankle inversion	Angular movement toward the medial plane.
	Medial longitudinal arch (MLA)	Located between the heel proximately and the medial three metatarsophalangeal joints anteriorly. Runs through metatarsals 1-3, sesamoid bones, cuneiform bones, navicular, talus, and calcaneus bones; the plantar aponeurosis, spring ligament, talocalcaneal ligament, and deltoid ligament; the flexor *hallucis longus*, flexor *digitorum longus, abductor hallucis*, flexor *digitorum brevis*, tibialis posterior.
	Ankle plantarflexion ROM in late stance	Refers to the angular distance of the movement around the ankle joint during the late stance of gait, characterized by a single limb support, and occurring before the swing period.
**Kinetic variables**	Initial peak vGRF	vGRF consists of the ground reaction force during walking, this is, the force exerted by the ground on the body (the sum of all forces that exist between a body and the supporting surface). Abnormal peaks or loading values can lead to overuse injuries. vGRF can be measured in different gait phases, like initial, breaking or propulsive stances. It's measured in Newtons (N).
	Breaking vGRF	
	Propulsive vGRF	
	Peak plantar pressure	Foot plantar pressure is described as the distribution of forces exerted in the field between the sole of the foot and the ground. It's measured in kilopascal (kPa).
	COP displacement (medio-lateral)	In biomechanics, COP is the specific point where the vGRF vector is applied. Its displacement consists of an oscillation, which might be identified in different axis of the body in relation to the ground.
	COP displacement (anterior-posterior)	

*ROM, range of motion; vGRF, vertical Ground Reaction Force; COP, center of pressure*.

This outcome includes foot posture and foot function assessments, which are relevant biomechanical measures, that can be extracted from the plantar pressure scans acquired previously.

Foot posture can be characterized using the modified arch index (MAI), which is calculated by the division of the foot length, minus the toes, in three equal portions, and also the division of pressure in the middle third by that of all three regions.

Foot function is characterized by the center of pressure excursion index (CPEI), which is a measure of foot function throughout the gait cycle, being defined as the distance between an imaginary line drawn from the first and last points of each foot's center of pressure (COP) trajectory and the COP as the distal third tertile of the foot. This value is usually normalized by foot width and multiplied by 100, to obtain a percentage excursion of the COP.

Following previous similar studies (7), CPEI and MAI's distribution are divided into quintiles. For the first, feet in the top and bottom quintile will be considered as having a supinated and pronated foot function, respectively, and for MAI, the top and bottom 20% will be considered *pes planus* and *pes cavus*, respectively.

Another variable of interest to characterize the podiatric profile is plantar loading characteristics. As there is no current consensus in the literature for the definition of adequate foot segments, this study will consider the templates of similar studies, which showed good reliability for plantar forces and pressures during barefoot walking in healthy adults (23). Namely, the regions to assess and compare are: lateral heel, medial heel, midfoot, 1st metatarsophalangeal joint (1MPJ), 2nd−5th metatarsophalangeal joint (2–5MPJ), hallux and the lesser toes.

The second primary outcome concerns the change in foot health at 5 months, a subjective evaluation by the participant, and is related to observed clinical parameters, signs and symptoms, namely skin, nails, foot structure, as well as presence and location of the pain. The Portuguese adaptation of the Self-Administered Foot Health Assessment Instrument (S-FHAI), a Likert-type instrument with four dimensions, will be used. It will be applied at month 0 and month 5. The majority of observed foot disorders will be recorded as either present or absent.

The study also includes the following secondary outcomes: (i) Foot Self-Care Knowledge, which is assessed between month 0 and month 5, through a four-dimensional questionnaire, where specific interventions for the promotion and prevention of foot and ankle disorders are evaluated; and (ii) Students' perceptions about the influence of foot health in their quality of life. It is evaluated through focus groups after exposure time (month 5).

Regarding potential effect modifiers and confounders, we expect that different nursing activities and ward typology (e.g., surgical units, intensive care) might produce diverse baropodometric patterns.

The primary and secondary outcomes, respective instruments for evaluation and timeframes for assessment are summarized in Table 2.

**Table 2:** Primary and secondary outcomes assessment.

			**Assessment**	**Timeframe**		
				**Month 0**	**Month 3**	**Month 5**
**Primary Outcomes**	Change in Podiatric Profile at 5 months^*^	Kinematic variables	EMED® software	x		x
		Kenetic variables		x		x
		Foot posture	MAI analysis	x		x
		Foot function	CPEI analysis	x		x
	Change in Foot Health at 5 months	Skin health	Portuguese adaptation of the self-administered foot health assessment instrument [S-FHAI; (24)].	x		x
		Nail health		x		x
		Foot structure		x		x
		Foot pain		x		x
**Secondary Outcomes**	Foot self-care knowledge	Skin: structure, problems and care	Portuguese adaptation of the Nurses' Foot Care Knowledge Test (NFKT)		X	
		Nails: structure, problems and care			X	
		Foot structural deformities: identification and care			X	
		Disease specific foot problems: identification and care			X	
		Footwear: properties and suitability			x	
	Students' Perceptions	Perceptions on the influence of clinical settings and potential foot disorders in quality of life.	Focal groups			x

* *global evaluation through the Emed® platform; MAI, Modified Arch Index; CPEI, Center of Pressure Excursion Index*.

### Study Layout

**Figure 1:**
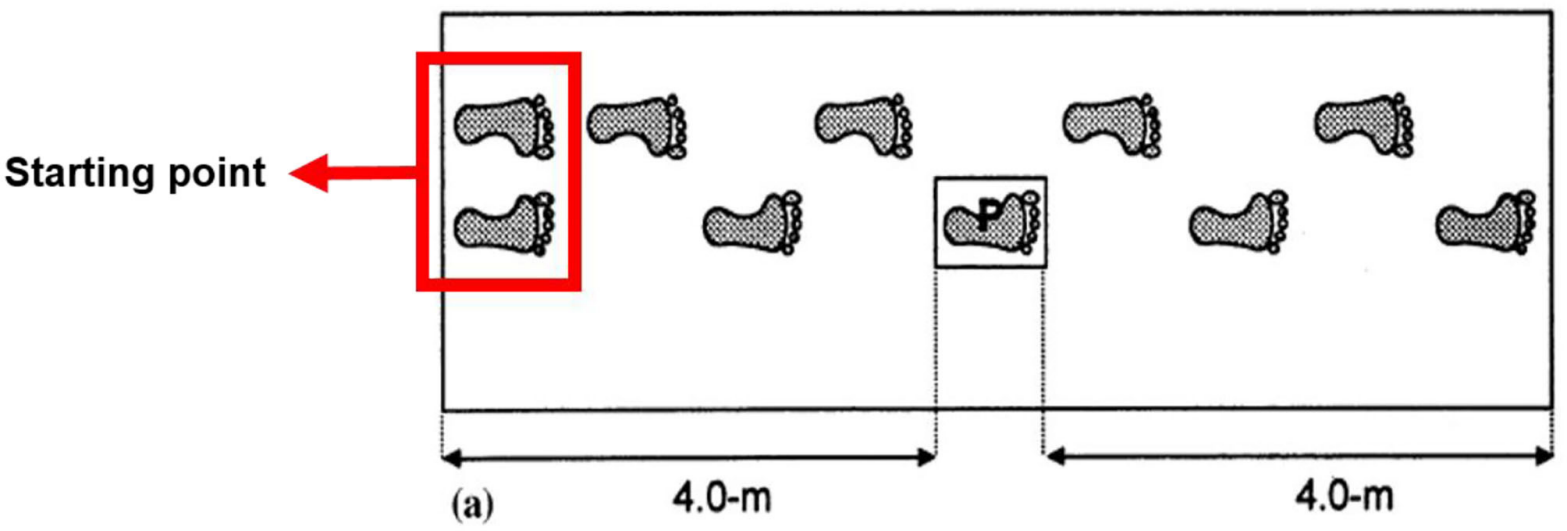
Midgait method. Participants start 4.0 m from the platform (P) [Adapted from Wearing et al. (25)].

After recruitment, at month 0 (before exposure) and month 5 (after exposure), the participants will undergo a four-phased procedure:

- Preparation: the trials will be explained to the participants and consent will be retrieved; any questions will be properly addressed;
- Familiarization: a test procedure will be performed where participants walk freely over the platform, without recording baropodometric gait analysis (BGA), to achieve the greatest possible freedom of movement. Participants will be asked to always walk barefoot.
- Trials: three types of gait protocols are usually applied in this type of study (one-step, two-step and mid-gait protocol). The choice should be based on both the variable of interest and the site of the foot to be studied. According to some authors (25, 26), there are few differences between the mid-gait (the *standard* protocol) and the two-step protocol, with similar values provided by both. An important criterion is using the same protocol throughout all studies, including follow-up.
  In this study, we chose to use the mid-gait protocol (Figure 1), particularly because the two-step termination protocol method is usually more appropriate for collecting joint kinematic data, which is out of the scope of this study, and also because the two-step initiation and termination protocols might delay acquiring the usual walking velocity during the trial (25, 26). In this sense, subjects will walk, at a self-selected pace, along an 8.0 m walkway. An initial position will be pre-established, to allow the right foot to hit the pedography platform at the middle of the walkway, with the fourth step, also avoiding gait alterations. After striking the platform, subjects will continue walking for 4.0 m until de end of the walkway.
  Additionally, participants will perform three valid trials per foot, as it has been previously found to be sufficient to ensure adequate reliability of force and pressure data (27–29) and will perform at least three steps before and after the pedography platform, as stated by Cousins et al. (27), that gait protocols should ideally involve a minimum preamble of at least three steps for a representative gait pattern to be obtained.
  It should be noted that a valid trial of a foot scan is defined as the participant hitting with the whole foot over the platform, without gait pattern alterations. A trial will be excluded, and should therefore be repeated, if a participant: (i) targets the borders of the platform with any part of the foot; (ii) alters gait to ensure full contact of the foot; and (iii) pauses on the mat.
- Self-Assessment of Foot Health: at the end of the BGA, participants will be asked to answer a self-assessment instrument about their current foot health, including skin, nail and presence of pain and will participate in a focus group together with another 5–10 participants from the study to discuss the influence of foot health in quality of life.

### Instruments

To record the desired data, the Emed^®^ platform from *novel.de* will be used. It consists of a pedography platform to measure pressure distribution under the foot, containing calibrated capacitive sensors, with the potential to accurately measure foot pressure in static and dynamic positions.

The main variables measured (throughout time) are: maximum pressures, pressure/time, force exerted, contact area and COP line.

According to the provider, the sensor platform has an area (mm) of 475 × 320 with a total of 6,080 sensors, with a resolution (sensor/cm^2^) of 4. The recording frequency is set at 50 Hz, with a pressure range (kPa) of 10–1.270. The pressure threshold is 10 kPa and the maximum total force is 193,000 N.

### Statistical Analysis

The primary outcomes and the Foot Self-Care Knowledge (secondary outcome) will be analyzed using SPSS Statistics v25, namely with descriptive statistics (average; standard deviation; medians) with an *a priori* significance value of *p* < 0.05.

After verifying the type of distribution of the sample, using the Kolmogorov-Smirnov (KS) with Liliefors correction (suitable for samples with more than 30 subjects) (30), either the independent samples *t*-test (normal distribution) or the Mann-Whitney (test non-normal distribution) will be used to compare changes in outcomes before and after the exposure.

To relate variables of interest, namely podiatric profile variables and specific foot-health parameters, a Multiple Linear Regression will be computed and multivariate analysis of variance with repeated measures to detect significant interactions between variables.

To control confounding effects, we'll perform a stratification of variables, and evaluate the exposure-outcome association, also using Mantel-Haenszel (M-H) estimator for an adjusted result. Additionally, the Analysis of Covariance (ANCOVA) will be computed to assess the effect of certain factors on the outcome variables.

The secondary outcome, related to the students' perceptions, will be analyzed using ATLAS.ti v7 software, based on the content analysis as defined by Bardin (31). The different phases of analysis are: (i) pre-analysis; (ii) exploration of the material; and (iii) treatment of results, inference and interpretation. The categorical organization of the content will be performed *a posteriori*, through the following codification steps: (i) cutting—choice of units; (ii) enumeration—choice of counting rules; and (iii) classification and aggregation of chosen categories and units.

Potential missing data will be handled using marginal mean imputation, this is, computing the mean of the missing value, *X*, using the non-missing values and use it to impute missing values of *X*.

Regarding loss to follow-up, all data belonging to participants that choose to opt-out or are lost during the study, will be erased from storage, as explained in the informed consent document.

## Discussion

We have described a protocol for a prospective observational cohort study, that aims to understand the influence of prolonged standing and walking positions on nursing students' foot health. This study will also explore the potential relationship between nursing students' podiatric profile and reported signs and symptoms.

Nursing practice is physically demanding, causing high loading forces, namely to the lower extremities. Additionally, common factors for increased risk for the development of foot/ankle disorders are footwear, constant standing and neglected foot care (6).

Currently, the study of injuries has been the subject of several studies, and some (31, 32) report that further investigations should be carried out into the circumstances and factors that cause problems at the level of foot/ankle region in this population.

Although several studies identify this problem, few describe in detail the causes of pain and discomfort. On the other hand, the podiatry evaluations conducted are poor (31), which limits a more detailed knowledge of the phenomenon under study. Moreover, and although some researchers suggest some solutions, namely the development of ergonomic and personalized footwear (33–35), there seems to be no adequate interventions to improve foot health in nursing students (4, 13).

Therefore, this study has the potential to comprehensively map significant foot/ankle changes caused by prolonged walking or standing positions, experienced by nursing students in clinical settings. Such data will be essential to establish a potential relationship between those factors. The conclusions will lead to a better understanding of the phenomena, thus allowing for better prevention. Additionally, the conclusions will lead to a better understanding of the phenomena, thus allowing for a better prevention, particularly through the development of guidelines and preventive tools.

Some limitations of the study are related to the convenience sample, which is recruited from a Nursing School in Portugal, reducing the external validity of the study. On the other hand, the follow-up time of 5 months, may be insufficient to elicit important signs and symptoms.

## Ethics Statement

The study was approved by the Ethical Committee of the Health Sciences Research Unit: Nursing (UICISA: E), of the Nursing School of Coimbra (ESEnfC), with the approval code no. P799_07_2021. The study was also recorded in ClinicalTrials.gov on the number NCT05197166. Participants will first be addressed in a project presentation session, where informed consent will be provided for their analysis and signature.

## Author Contributions

RB, SC, and AG led the writing of the original protocol. PP, LS, and PS-C contributed to the statistical analysis section. RB, SC, AG, IA, and FP-S contributed to the qualitative analysis section. RB, SC, and PS-C were responsible for the final review of written English. PP, RB, and AG were responsible for the recruitment and exclusion/inclusion criteria sections and initially wrote a draft of the study layout, which was later validated and corrected by SC, LS, FP-S, and IA. RB, SC, PP, IA, and AG outlined the major outcomes of interest and, with the contribution of LS and PS-C, described them for the present study. The global administration of the project is led by RB, SC, and AG.

## Funding

This work is part of the Ph.D. project of the RB, funded by the FCT—Foundation for Science and Technology, I. P., through the grant number UI/BD/151102/2021. It is also funded by National Funds through the FCT—Foundation for Science and Technology, I. P., within the scope of the project reference number UIDB/00742/2020 and project reference number UIDB/04279/2020.

## Conflict of Interest

The authors declare that the research was conducted in the absence of any commercial or financial relationships that could be construed as a potential conflict of interest.

## Publisher's Note

All claims expressed in this article are solely those of the authors and do not necessarily represent those of their affiliated organizations, or those of the publisher, the editors and the reviewers. Any product that may be evaluated in this article, or claim that may be made by its manufacturer, is not guaranteed or endorsed by the publisher.
